# Gender-specific association of serum uric acid levels and cardio-ankle vascular index in Chinese adults

**DOI:** 10.1186/s12944-018-0712-x

**Published:** 2018-04-11

**Authors:** Xiaoya Zheng, Qiang Wei, Jian Long, Lilin Gong, Hua Chen, Rong Luo, Wei Ren, Yonghong Wang

**Affiliations:** 1 Department of Endocrinology, The First Affiliated Hospital of Chongqing Medical University, No.1 Friendship Road, Yuzhong District, Chongqing, China; 2 The Public Health Center, The First Affiliated Hospital of Chongqing Medical University, No.1 Friendship Road, Yuzhong District, Chongqing, China; 3 School of Public Health and Management, Chongqing Medical University, Chongqing, China

**Keywords:** Serum uric acid, Hyperuricemia, Cardio-ankle vascular index, Cardiovascular disease, Chinese

## Abstract

**Background:**

Little is known about the relationship between serum uric acid (SUA) and cardio-ankle vascular index (CAVI) in Chinese population. Therefore, we aimed to investigate the gender difference in the association of SUA and CAVI in a southwestern Chinese population.

**Methods:**

Data were obtained from subjects via routine physical examinations in the Public Health Center of our hospital between 2011 and 2014 in Chongqing. The data included completed anthropometry and blood biochemical indicators. The CAVI were recorded using an automatically VaseraVS-1000 vascular screening system.

**Results:**

We found females with hyperuricemia (HUA) had significantly higher CAVI than women with normal SUA (8.45 ± 1.40 vs 7.67 ± 1.15, P<0.05). Then we defined high CAVI as CAVI≥9 m/s, and compared the percentage of high CAVI, we found women with HUA had higher percentage of high CAVI than women with normal SUA (26.83% vs 9.38%, P<0.05). Those differences were not significant in males. Also, the logistic regression analysis found age and hypertension were major independent risk factors associated with high CAVI in both genders. HUA and hyperglycemia were independently associated with high CAVI in females with an OR of 3.65, 95%CI (1.37-9.73) and 3.02, 95%CI (1.38-6.63) respectively. However, these significant associations were not be found in males.

**Conclusions:**

Our data showed positive associations between elevated SUA levels and higher CAVI risk in the inland Chinese females, but not in males. The reason for the gender differences were still unclear, sex hormones may play a role. Further prospective studies including detailed personal information and multicenter were required.

## Background

Arterial stiffness has been established as an independent predictor for the prognosis of cardiovascular disease [1]. Arterial stiffness can be measured by several methods including brachial-ankle pulse wave velocity (baPWV), augmentation index (AIx), beta stiffness index and the relatively new method of cardio-ankle vascular index (CAVI) [2]. The most outstanding feature of the CAVI is the lack of dependence on blood pressure at the time of measurement [3]. Recent studies [4–6] showed that arterial stiffness evaluated by the CAVI is an independent predictor of cardiovascular events in patients with intermediate atherosclerotic risk factors, and an increase CAVI was associated with a significant excess mortality. These findings indicated the importance of arterial stiffness as a strong predictor of cardiovascular outcome among several candidate predictors.

Serum uric acid (SUA), the end product of purine metabolism, also has been linked to both metabolic syndrome (MetS) and cardiovascular disease [7]. Several epidemiological studies [8–10] have suggested that increased SUA is an independent predictor of cardiovascular disease and mortality. Additionally, epidemiological study has indicated that the risk of cardiovascular mortality related to increase SUA is greater in women than in men [11]. Recently, a study [12] in Japanese population found an independent correlation between SUA and CAVI, and observed gender difference in the SUA range for increase in CAVI. However, the relationship between SUA and CAVI has not been reported in Chinese population.

In this study, we intended to investigate whether SUA is associated independently with CAVI in Chinese adults. Additionally, we also explored the gender difference in the relationship between SUA and CAVI.

## Methods

### Study population

Data were obtained from subjects who underwent routine health examination in the Public Health Center of the First Affiliated Hospital of Chongqing Medical University from January 2011 through December 2014. Exclusion analysis criteria: 1) Subjects taking antihypertensive agents (especially diuretics, such as furosemide), anti-diabetic agents, lipid-lowing agents, or hypouricemic agents (including drugs that inhibit uric acid synthesis, such as allopurinol, and drugs that promote uric acid excretion, such as sulfinpyrazone, probenecid and benzbromarone); 2) other known or diagnosed chronic diseases such as stroke, hypertension, diabetes, dyslipidemia, heart disease, chronic liver disease, and gout. 3) Subjects with incomplete data were excluded for analyzing to avoid bias. At last, 1217 subjects (696 men and 521 women) were analyzed in this study. The study was approved by Ethics and Human Subject Committee of Chongqing Medical University. Given the retrospective nature of the study, we were granted a waiver of informed consent.

### Anthropometric measurements

Anthropometric measurements included height, weight, waist circumference, and blood pressure. Height and weight were measured while the subjects were wearing light clothing and no shoes. BMI was calculated as weight (kg) divided by the square of the height (m). Waist circumference was measured at the midpoint between the bottom of the rib cage and the top of the iliac crest at the end of exhalation. Blood pressure, including systolic blood pressure (SBP) and diastolic blood pressure (DBP), was measured using each subject’s right arm after a 5 min rest and in a sitting position.

### Assessment of biomarkers

Blood biochemical analyses included serum uric acid (SUA), cholesterol (TC), triglycerides (TG), low-density lipoprotein cholesterol (LDL-c), high-density lipoprotein cholesterol (HDL-c), and fasting plasma glucose (FPG). These indicators were measured by an inmmuno-chemical-automated analyzer (Type 7600, Hitachi Ltd., and Japan).

### Assessment of CAVI and ABI

CAVI was recorded using a VaseraVS-1000 vascular screening system (Fukuda Denshi, Tokyo, Japan) with the participants resting in a dorsal position. Electrocardiograph electrodes were placed on both wrists, a microphone for detecting heart sounds was placed on the sternum, and cuffs were wrapped around both the arms and ankles. After automatic measurements, obtained data were analyzed by software, and the value of CAVI was obtained automatically. For statistical evaluation of the CAVI, mean values of the left and right sides were used.

### Definitions

High CAVI was defined as CAVI≥9 m/s [13] . Older age was defined as age ≥ 65y. Hypertension (HBP) was defined as SBP ≥140 mm Hg and/or DBP ≥90 mm Hg, and/or the current use of antihypertensive medication [14]. Overweight was defined as BMI ≥ 25Kg/m^2^ [15]. Hyperglycemia was defined as fasting blood glucose (FBG) ≥ 6.1 mmol/L. High LDL-c was defined as LDL-c ≥ 2.6 mmol/L). High TG was defined as TG ≥ 1.7 mmol/L. Low HDL-c was defined as HDL-c < 0.90 mmol/L in men or < 1.00 mmol/L in women [16]. Hyperuricemia (HUA) was defined as SUA > 420 μmol/L (7.0 mg/dL) for men and > 360 μmol/L (6.0 mg/dL) for women [17].

### Statistical analyses

Statistical analyses were performed using SAS software (version 9.0; SAS Institute, Inc., Cary, North Carolina). Continuous variables with normal distributions are expressed as the mean ± standard deviation ($\overline{X}$±SD), and categorical variables are described as percentages (%). Multiple-group comparisons of means were performed using generalized linear models (GLMs). Chi-square tests were used to compare percentages. Logistic regression was used to obtain odds ratios (ORs) of categorical variables. All statistical analyses were two-sided, and *p*-values less than 0.05 were considered to be statistically significant.

## Results

**Fig. 1:**
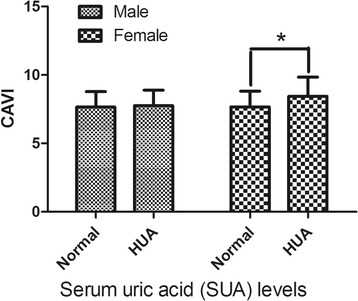
CAVI levels of males and females with and without hyperuricemia (HUA). HUA is defined as SUA > 420 μmol/L in male, and > 360 μmol/L in female. * means *P* < 0.05 females with HUA compared with females with normal uric acid. Abbreviations: CAVI, cardio-ankle vascular index; HUA, hyperuricemia

In total, 1217subjects (696 men and 521 women) were analyzed in this study. Hyperuricemia (HUA) is defined as SUA > 420 μmol/L in male, and > 360 μmol/L in female. The prevalence of HUA in male is 25.1%, and in female is 7.9%. Subjects with HUA have a higher age, BMI, waist, SBP, DBP, TG, and FPG in both men and women. Only HDL-c levels were lower in subjects with HUA than in controls. The TC and LDL-c in HUA and control groups had no significant difference in both men and women. Men with HUA did not have significantly higher CAVI than men with normal uric acid (7.75 ± 1.15 vs 7.66 ± 1.12, *P*>0.05), while women with HUA had significantly higher CAVI than controls (8.45 ± 1.40 vs 7.67 ± 1.15, *P*<0.05). Details are shown in Table 1 and Fig. 1.

**Table 1:** Demographic and laboratory variables of the analyzed men and women

Factors	Male		Female	
	Control	HUA	Control	HUA
*N* (%)	521 (74.9)	175 (25.1)	480 (92.1)	41 (7.9)
Age (y)	48.2 ± 11.4	50.0 ± 11.3*	53.5 ± 10.6	56.6 ± 10.5*
BMI (Kg/m2)	24.4 ± 2.9	25.5 ± 3.0*	23.7 ± 3.0	25.8 ± 3.9*
WC(cm)	84.3 ± 8.0	88.3 ± 8.3*	77.0 ± 7.6	83.0 ± 11.0*
SBP (mmHg)	128.0 ± 19.2	131.8 ± 18.2*	127.4 ± 20.2	139.4 ± 25.6*
DBP (mmHg)	79.8 ± 12.6	84.6 ± 13.5*	75.0 ± 11.4	79.6 ± 11.7*
TC (mmol/L)	5.1 ± 1.0	5.3 ± 1.0	5.2 ± 1.0	5.3 ± 1.4
TG (mmol/L)	1.8 ± 1.6	2.8 ± 1.8*	1.4 ± 1.2	2.0 ± 1.3*
HDL-c(mmol/L)	1.5 ± 0.4	1.3 ± 0.3*	1.7 ± 0.4	1.5 ± 0.3*
LDL-c(mmol/L)	3.0 ± 0.8	3.1 ± 0.8	2.9 ± 0.9	3.0 ± 1.0
FPG(mmol/L)	5.2 ± 1.6	5.5 ± 1.5*	5.1 ± 0.9	5.3 ± 1.2*
CAVI	7.66 ± 1.12	7.75 ± 1.15	7.67 ± 1.15	8.45 ± 1.40*

*Abbreviations: BMI* body mass index, *CAVI* cardio-ankle vascular index, *DBP* diastolic blood pressure, *FPG* fasting plasma glucose, *HDL-c* high density lipoprotein-cholesterol, *HUA* hyperuricemia, *LDL-c* low-density lipoprotein-cholesterol, *SBP* systolic blood pressure, *SUA* serum uric acid, *TC* total cholesterol, *TG* triacylglycerol, *WC* waist circumference

*P < 0.05 compared with control

**Fig. 2:**
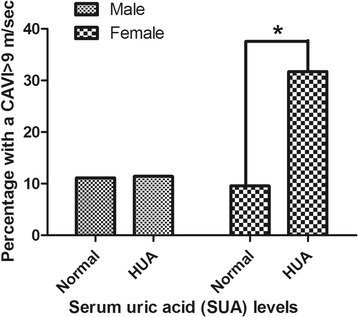
Percentage of high CAVI in males and females with and without hyperuricemia (HUA). HUA is defined as SUA > 420 μmol/L in male, and > 360 μmol/L in female. * means *P* < 0.05 females with HUA compared with females with normal uric acid. Abbreviations: CAVI, cardio-ankle vascular index; HUA, hyperuricemia

The CAVI is considered to be a diagnosis of atherosclerosis when it is greater than or equal to 9 m/s [13]. So high CAVI is defined as CAVI≥9 m/s, and then we compared the percentage of high CAVI in both genders. We found there was no significant difference of percentage of high CAVI between HUA and control groups in male (11.43% vs 11.13%, *P*>0.05). While women with HUA had a significantly higher percentage of high CAVI than women with normal uric acid levels (26.83% vs 9.38%, *P*<0.05). Details are shown in Fig. 2.

**Fig. 3:**
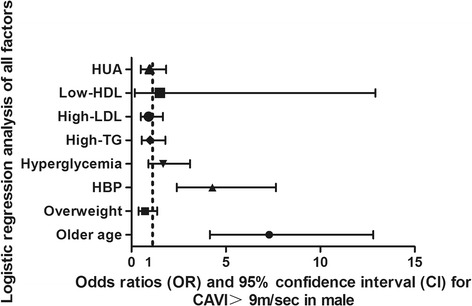
Logistic regression analysis of all risk factors of high CAVI in males. Abbreviations: CAVI, cardio-ankle vascular index; HBP, High blood pressure; HDL, high density lipoprotein; HUA, hyperuricemia; LDL, low-density lipoprotein. High CAVI was defined as: 0 for normal CAVI < 9 m/s, 1 for high CAVI≥9 m/s. Older age, HBP, overweight, hyperglycemia, high-LDL-c, high-TG, low-HDL, and HUA (both classified as yes or no) were coded as: 0 for no, 1 for yes

High CAVI (yes or no) was introduced as a dependent variable in the multiple factors logistic regression analysis models, older age, HBP, overweight, hyperglycemia, high-LDL-c, high-TG, low-HDL, and HUA (both classified as yes or no) as independent variables. In this model of male subjects, higher age and HBP were risk factors for high CAVI, with an OR of 7.30, 95%CI (4.16-12.8), and 4.29, 95%CI (2.40-7.66), respectively. After adjusting for these risk factors, HUA was not an independent risk factor for high CAVI, with an OR of 0.96, 95%CI (0.5-1.84). Details are shown in Fig. 3.

**Fig. 4:**
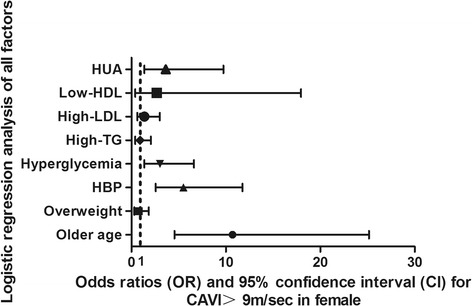
Logistic regression analysis of all risk factors of high CAVI in females. Abbreviations: CAVI, cardio-ankle vascular index; HBP, High blood pressure; HDL, high density lipoprotein; HUA, hyperuricemia; LDL, low-density lipoprotein. High CAVI was defined as: 0 for normal CAVI < 9 m/s, 1 for high CAVI≥9 m/s. Older age, HBP, overweight, hyperglycemia, high-LDL-c, high-TG, low-HDL, and HUA (both classified as yes or no) were coded as: 0 for no, 1 for yes

In this model of female subjects, higher age with an OR of 10.71, 95%CI (4.66-25.16), HBP with an OR of 5.50, 95%CI (2.58-11.76), and IFG with and OR of 3.02, 95%CI (1.38-6.63) were risk factors for high CAVI. After adjusting for these risk factors, HUA was also an independent risk factor for high CAVI, with an OR of 3.65, 95%CI (1.37-9.73). Details are shown in Fig. 4.

## Discussion

In the present study, we found a significantly gender-specific association of serum uric acid levels and CAVI in Southwest Chinese adults. Females with HUA had significantly higher levels of CAVI and higher percentage of high CAVI than females with normal SUA. Those differences were not significant in males. Also, the logistic regression analysis found age and HBP were major independent risk factors associated with high CAVI in both genders. After adjusting for age and blood pressure, HUA and hyperglycemia were independently associated with high CAVI in females, but not in males.

We were interested to the result that the associations of SUA with CAVI were stronger in women than in men. Consistent with our results, many studies had found that SUA levels are predictive of cardiovascular disease among women, but not men. Recently study [18] found SUA is associated with cardiac diastolic dysfunction among women with preserved ejection fraction, but not in men. Masayuki et al. reported an increased level of SUA was associated with major cardiovascular adverse events (MACE) more strongly in women than in men with acute coronary syndrome (ACS) [19]. Chou et al. reported that the SUA level was associated with insulin resistance and plasma glucose levels more strongly in non-diabetic women than in men [20]. Gender-related differences were also be found in the association of SUA and arterial stiffness in patients with hypertensive chronic kidney disease [21], the link between SUA and left ventricular mass index in patients with obstructive hypertrophic cardiomyopathy [22], and the relationship between SUA and cardiac hypertrophy in patients with chronic kidney disease [23]. A study [12] in Japanese population found the higher SUA was independently associated with high CAVI in both genders, but the SUA range for increase in CAVI was lower in females than in males. Also in a Japanese population, Ishizaka et al. showed that Pearson’s correlation between the SUA level and brachial-ankle pulse wave velocity (baPWV) is significant in females, but not in males [24]. Although the reasons for these gender differences were still unclear, sex hormones may play a role.

It is well known that the SUA levels in male were higher than in female due to some gender related factors. The apparently protective effect of female might be related to estrogen level. Estrogen is known to possess the effect of promoting excretion of uric acid. Here, the sex difference in the risk of hyperuricemia may lie in the fact that estrogen is a uricosuric agent [25]. A previous study by Anton et al. showed that higher renal clearance of urate in women was due to their higher plasma estrogen levels and lower tubular urate post secretory reabsorption [26]. A recent study by Liu et al. showed for males, eating habits have greater influences on the risk of developing hyperuricemia. For females, lifestyle factors like work type, commuting method, and exercise have such effects [27].

Sex hormone and lifestyle differences may partly explain the different risk of HUA in males and females. However, it remains unclear why the association between HUA and high CAVI differs between males and females. Chou et al. hypothesized that women with HUA have a relatively increased burden of hyper-insulinemia and hyperglycemia, and they proposed that the SUA level is more important for predicting the degree of insulin resistance in women than in men. In addition the significant sex differences in the relationships of serum uric acid with insulin resistance may explain part of this stronger association between SUA and coronary artery disease in women [20]. Thus, the future studies to explain the exact mechanism of gender specific association of SUA and CAVI are still needed.

The exact mechanisms by which HUA increases arterial stiffness remain unknown. Several mechanisms may link SUA and arterial stiffness. First, SUA promotes proliferation, angiotensin II production, and oxidative stress in vascular smooth muscle cells via the tissue renin-angiotensin system [28, 29]. Second, HUA decreases the production of nitric oxide in vascular endothelial cells and induces endothelial dysfunction and decreases flow-mediated vasodilation [30]. Third, xanthine oxidase that catalyzes the oxidation of xanthine and hypoxanthine to uric acid may also contribute to impaired endothelial function by producing superoxide anions [31, 32]. Therefore, it is still uncertain whether uric acid is a pathogenic mediator or just a marker of atherosclerosis.

There are several potential limitations of our study. The main limitation is the lack of information on lifestyle, smoking and diet, which may be helpful to understand the relationship between CAVI and SUA levels. We infer that smoking status might be an important confounder in analyze the relationship between CAVI and SUA, especially in male adults. Further studies including detailed personal information were required. Second, this study is based on a retrospective analysis, though we found significant association between SUA and CAVI in females, we could not investigate the exact pathophysiology relationship between SUA and CAVI. Further prospective study on SUA and CAVI is necessary. Third, most of the subjects included in this study were residents of inland Chongqing, due to different lifestyle and eating habits of inland and coastland residents, the association of CAVI and SUA levels might not be the same. Further multicenter studies may be helpful.

## Conclusions

In conclusion, our data showed positive associations between elevated SUA levels and high CAVI risk in the inland Chinese females, but not in males. The reason for the gender differences were still unclear, sex hormones may play a role. Further prospective studies including detailed personal information and multicenter were required.

## Abbreviations

ACS: Acute coronary syndrome
Aix: Augmentation index
baPWV: Brachial-ankle pulse wave velocity
BMI: Body mass index
CAVI: Cardio-ankle vascular index
CI: Confidence interval
DBP: Diastolic blood pressure
GLMs: Generalized linear models
HBP: Hypertension
HDL-c: High density lipoprotein-cholesterol
HUA: Hyperuricemia
IR: Insulin resistance
LDL-c: Low-density lipoprotein-cholesterol
MACE: Major cardiovascular adverse events
MetS: Metabolic syndrome
ORs: Odds ratios
SBP: Systolic blood pressure
SD: Standard deviation
SUA: Serum uric acid
TC: Total cholesterol
TG: Triacylglycerol
WC: Waist circumference
